# Biological Functions and Applications of Virus-Related Bacterial Nanoparticles: A Review

**DOI:** 10.3390/ijms23052595

**Published:** 2022-02-26

**Authors:** Toshiki Nagakubo

**Affiliations:** 1 Department of Life and Environmental Sciences, University of Tsukuba, Tsukuba 305-8577, Japan; nagakubo.toshiki.gp@u.tsukuba.ac.jp; 2 Microbiology Research Centre for Sustainability (MiCS), University of Tsukuba, Tsukuba 305-8577, Japan

**Keywords:** phage tail-like nanostructures, eCIS, biological interactions, bacteriophages, nanomedicines, structure and functional mechanism

## Abstract

Accumulating evidence suggests that microorganisms produce various nanoparticles that exhibit a variety of biological functions. The structure of these bacterial nanoparticles ranges from membrane vesicles composed of membrane lipids to multicomponent proteinaceous machines. Of bacterial nanoparticles, bacterial phage tail-like nanoparticles, associated with virus-related genes, are found in bacteria from various environments and have diverse functions. Extracellular contractile injection systems (eCISs), a type of bacterial phage tail-like nanostructure, have diverse biological functions that mediate the interactions between the producer bacteria and target eukaryote. Known gram-negative bacterial eCISs can act as protein translocation systems and inject effector proteins that modulate eukaryotic cellular processes by attaching to the target cells. Further investigation of the functions of eCISs will facilitate the application of these nanomachines as nano-sized syringes in the field of nanomedicine and vaccine development. This review summarises the recent progress in elucidating the structures and biological functions of nanoparticles that resemble the tail components of phages that infect bacteria and discusses directions for future research to improve the clinical applicability of virus-related bacterial nanoparticles.

## 1. Introduction

Bacteria produce various types of nanoparticles with versatile functionalities. It has been revealed that the biogenesis of some bacterial nanoparticles is closely associated with virus-related genes that are conserved among many classes of bacteria [1,2,3,4,5]. While the origins of these viral genes and the mechanism by which these genes are acquired by bacteria are not fully understood, virus-related bacterial nanoparticles have great potential for application in diverse fields of science. In this review, I focus on the current knowledge of the biological functions of bacterial phage tail-like nanostructures. Phages are viruses that infect bacteria. Phages belonging to the order *Caudovirales* comprise typical structural components, namely the head and tail, and there are two types of typical tail structures of these phages: rigid tails and flexible tails, exemplified by myophages and siphophages, respectively [4]. While both types of phage tails are involved in the translocation of the phage genome, rigid phage tails show a contractile mechanism of action in which the tail tube inside the tail sheath lumen of the tail is ejected upon contraction of the tail sheath [4]. Many bacterial genomes contain gene clusters encoding structural proteins homologous to those of phage tails and proteins related to the phage infection cycle [3,6,7]. The macromolecular structures of the products of these gene clusters are structurally similar to the tail components of phages; however, these phage tail-like nanostructures are not infectious because of the lack of head components that accommodate the viral genome. Recent studies have revealed a variety of biological functions of these nanostructures, including their intriguing roles in mediating eukaryote–bacteria interactions.

In the following sections, I summarise recent advances in elucidating the structures and biological functions of these bacterial nanoparticles, especially focusing on extracellular contractile injection systems (eCISs) that are widely distributed among microorganisms existing in various environments [6]. eCISs are distinct from other phage tail-like nanostructures in that they are released from the producer cells and act as protein translocation systems by attaching to the target cells [8]. Their functions have attracted considerable interest not only in biological aspects but also in the fields of biotechnology, medicine and vaccine development because of their potential as nano-sized syringes injecting various types of effector proteins into the target cells [8,9].

## 2. Intracellular and Extracellular Phage Tail-like Nanostructures

**Figure 1:**
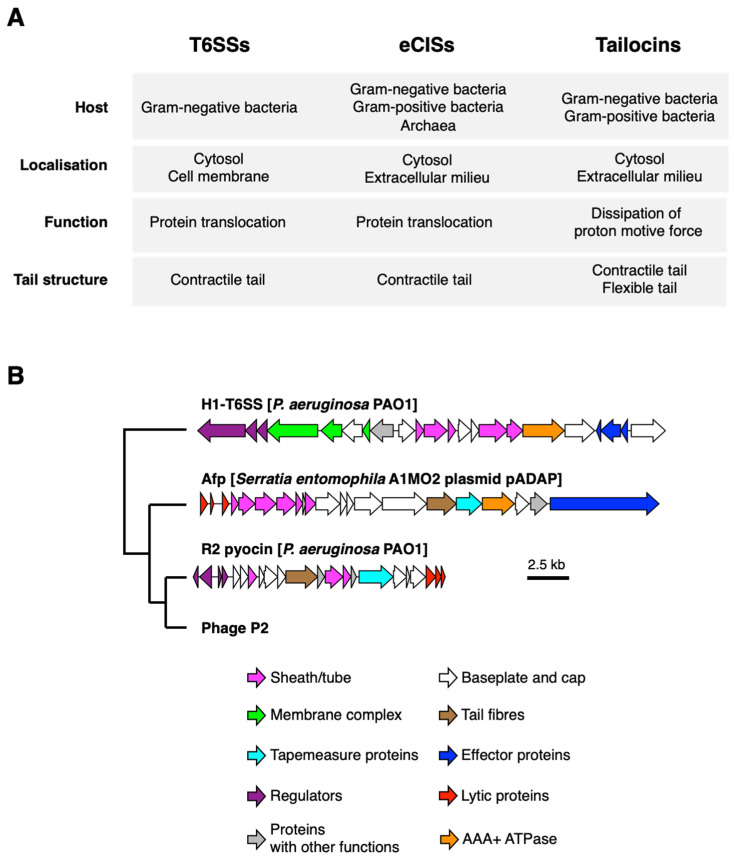
Features and genetic compositions of three classes of phage tail-like nanomachines. (**A**) Features of three classes of phage tail-like nanomachines are listed. (**B**) Genetic compositions of gene clusters encoding phage tail-like nanomachines, and their phylogenetic relationship are shown [10,11,12]. The phylogenetic relationship is based on that reported in a previous study [6].

Bacterial phage tail-like nanostructures are currently classified into three groups—type VI secretion systems (T6SSs), tailocins and extracellular injection systems (eCISs)—based on several features, including amino acid sequences and localisation [6,10] (Figure 1A).

T6SSs are nanomachines whose components have been proposed to be evolutionarily related to contractile bacteriophage tails [13]. T6SS are encoded by a cluster of 15–20 genes present in at least one copy in approximately 25% of all sequenced gram-negative bacteria, especially Proteobacteria [13]. T6SSs are cell-envelope-spanning nanomachines that, upon contact with the target cell, puncture their tail tube and inject toxic proteins called effectors into the target cells across the cell envelope through a phage-tail-like contraction mechanism, killing the cell [14]. T6SSs were originally discovered in pathogenic bacteria mediating pathogen-host interactions by translocating toxic effectors into target cells [15]. To date, among the known T6SS functions, their contributions to inter-bacterial competition have been extensively studied [16,17,18,19,20,21,22] and reviewed at numerous instances [3,23,24]. The T6SS is composed of at least 13 conserved essential core components and several accessory components [24]. The machinery consists of two main complexes—a contractile phage tail-like complex and a membrane complex comprising inner membrane proteins. Three proteins, namely, TssJ, TssL and TssM, are minimal components of the membrane complex [25]. The contractile phage tail-like complex is comprised of the Hcp tube, VgrG spike with tip protein and TssB-TssC sheath [24]. Although T6SSs and other classes of phage tail-like nanostructures have a similar mechanism of action, they have unique features. For example, T6SS functions via the AAA+ ATPase. ClpV AAA+ ATPase is responsible for disassembling the contracted sheath, thereby restoring the pool of available sheath subunits [24]. This is different from the role of AAA+ ATPase in eCISs, in which AAA+ ATPase is essential for protein assembly [26].

In contrast to T6SSs, tailocins and eCISs are released from the producer cells and act in the extracellular environment. Although the structures of tailocins and eCISs share several features with T6SS structures, they are more closely related than T6SSs to contractile phages in terms of their localisation and mechanisms of action [6]. These nanostructures are believed to be released into the extracellular milieu from the producer cells via cell lysis [27]; they puncture the cell envelope of the target cell following their attachment to the cell surface [28]. Gene clusters encoding these extracellular phage tail-like nanostructures often include viral lytic genes encoding endolysins and holins that can mediate cell lysis (Figure 1B). In some cases, this process is triggered by conditions that induce phage induction [29]. Temperate phages are bacterial viruses that have a dual life cycle—lysogenic and lytic cycles [30]. In the lysogenic cycle, phage DNA integrates into the bacterial genome as a prophage, and the induction of prophage initiates lytic replication in which lytic proteins and structural proteins are expressed [31]. The expression of phage genes is induced by certain stresses, such as DNA damaging stress, to host cells [30]. Endolysins and holins are typical viral proteins involved in programmed host cell lysis, by which the newly synthesised phage particles are released from the cells. Endolysins are muralytic enzymes that often contain cell-wall-binding domains, such as LysM domains [32]. Holins are small membrane proteins that form pores, through which endolysins can access the peptidoglycan on the cytosolic membrane of the host cells [32]. Holins interact with antiholins, which inactivate holins until lysis triggering [32]. Once these lytic proteins are expressed and lytic processes are initiated, they degrade the cell wall of the host cells, which, in turn, cause cell lysis because of the weakened cell wall integrity [32]. As endolysin/holin pairs are often found in gene clusters encoding tailocins and eCISs, these viral lytic processes are considered to be involved in the release of these phage tail-like nanostructures [33,34].

## 3. Tailocins

Tailocins are bactericidal phage tail-like particles that are believed to attach to the target cell and inhibit cell growth by penetrating the cell envelope and dissipating proton motive force [35]. Typically, tailocins target bacteria closely related to the producer strain. *Pseudomonas aeruginosa* pyocins, which are well-characterized tailocins, inhibit the growth of some related strains, and the target specificities differ among different producer strains [11,36]. This indicates that they have evolved to specifically attack certain competitors, and/or some strains have acquired resistance against these pyocins. Tailocins are subdivided into the R-type and F-type based on their tail morphology [28]. R-type tailocins have a rigid and contractile tail that ejects the cell-puncturing tail tube by contracting the tail sheath accommodating the tube, while F-type tailocins have a non-contractile flexible tail, and their mechanism of action remains largely unknown [28]. R-type and F-type tailocins are suggested to have evolved from P2 phages and lambda phages, respectively, both of which are temperate enterobacteria phages originally isolated from *Escherichia coli* [28]. Recently, the cryo-EM analysis revealed an atomic model of R-type pyocin in its pre-contraction and post-contraction states [11]. From these structures, the contraction mechanism of R-type pyocins at the molecular level has been proposed [11].

## 4. Extracellular Contractile Injections Systems (eCISs)

### 4.1. Distribution and Evolution of eCISs

Although eCISs, which comprise another group of extracellular phage tail-like nanostructures, are structurally similar to R-type tailocins [11,28], they are likely to be evolutionarily distant from each other. Sarris et al. examined the evolutionary relations between eCISs, T6SSs, R-type pyocins and T4/P2 phages [6]. Each class of these nanostructures forms a monophyletic group, suggesting that these phage-related nanostructures have evolved independently from their common ancestor. The authors have also identified a minimal set of genes comprising the consensus eCIS gene cluster and compared them with the orthologs of T6SSs, R-type pyocins and phages [6]. One of the distinct features of the eCIS gene clusters is the absence of genes encoding helicase and TssJLM homologs that are conserved in T4/P2 phages and T6SSs, respectively [6], suggesting that eCISs are not simple defective phages, and that eCISs, T6SSs and phages have diversified by the acquisition and/or loss of these genes in bacteria [6] (Figure 1B). Although the composition of the structural genes of the R-type pyocin gene cluster is relatively similar to that of eCISs, the presence of AAA+ ATPase is a critical feature of eCIS gene clusters [6] (Figure 1B). In addition, the reconstruction of the phylogeny of eCIS-related genes has also led to the idea that extensive horizontal gene transfer events might have occurred during the spread of eCISs amongst microorganisms [6,10]. No full agreement has been established between the phylogeny of eCIS subunit proteins and the phylogeny of the bacteria carrying eCIS gene clusters [6]. Additionally, some eCIS-related gene clusters are carried on plasmids, implying their role in the horizontal gene transfer of eCISs across species [6].

eCIS-related genes can be widely found in the sequenced genomes of many bacterial species belonging to the following phyla: Acidobacteria, Actinobacteria, Chlorobi, Chloroflexi, Cyanobacteria, Deinococcus-Thermus, Firmicutes, Gemmatimonadetes, Nitrospirae, Verrucomicrobia, Enterobacteria, Proteobacteria and Bacteroidetes [6,10]. Moreover, notably, eCIS-related genes can be found in some archaeal species [6,10]. Chen et al. classified 631 eCIS-like gene clusters, which were extracted from 11,699 publicly available complete bacterial genomes, into six classes (Ia, Ib, IIa, IIb, IIc and IId) based on their genetic composition and organisation [10]. These classes are different from each other in terms of the number of sheath/tube proteins, the presence/absence of Afp13-like tail fibre protein and tape measure protein. Interestingly, the authors have also reported that eCIS and T6SS loci are rarely found encoded in the same genome and proposed the following three possibilities to explain this observation: (i) the similarity of shared subunits, such as VgrG spike protein, cause misassembly in these protein machines, (ii) these two systems fulfil the same biological role and (iii) the expression of their structural proteins have a large metabolic cost that could potentially impose significant selective pressure on the producer bacteria [10].

### 4.2. Biological Functions of eCISs

#### 4.2.1. Gram-Negative Bacterial eCISs

**Figure 2:**
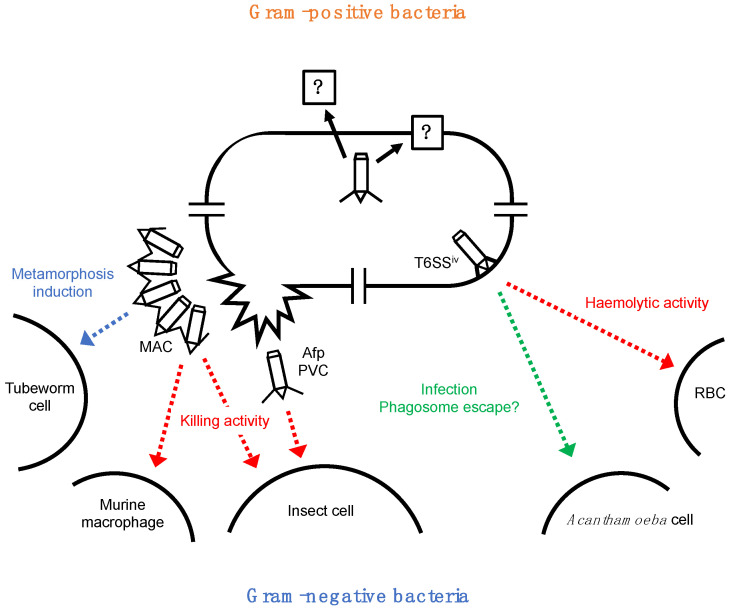
Currently known functions of eCIS: a schematic model of the localisation and biological functions of known eCISs and related nanostructures. MAC, metamorphosis-associated contractile structures. Afp, anti-feeding prophage. PVC, *Photorhabdus* virulence cassette. RBC, red blood cell.

Amongst eCISs conserved across bacterial species, gram-negative bacterial eCISs are the most functionally characterised nanomachines, which exhibit unique bioactivities that mediate prokaryote-eukaryote interactions (Figure 2).

Anti-feeding prophage (Afp) is a phage tail-like contractile injection system isolated from *Serratia entomophila*, a causal agent of amber disease in the New Zealand grass grub *Costelytra zealandica*. Amber disease has a distinct phenotypic progression wherein the infected host stops feeding within 2 to 4 days of ingesting pathogenic bacteria [37]. At this time, levels of the major digestive enzymes decrease sharply and the normally black-grey gut clears, resulting in a characteristic amber colour of the infected insects [37]. The infected larvae may remain in this state for a prolonged period (1 to 3 months) before the infecting bacteria eventually invade the haemocoel, causing death [37]. In 1993, two regions of the 153-kb plasmid pADAP were identified as necessary for causing amber disease symptoms [38]. The sep-virulence-associated region comprises three genes, sepABC, which are responsible for the symptoms of gut clearance and amber colouration of the larvae [39]. Transposon insertion in any of the sep genes abolished gut clearance but not the cessation of feeding, indicating the presence of an anti-feeding gene(s) elsewhere in pADAP [39]. In 2004, Hurst et al. reported that a putative defective prophage in pADAP is essential for amber disease symptoms, including cessation of feeding, clearance of the gut and amber colouration of the larvae (Figure 2) [40]. The authors named the prophage region and its product as Afp; they showed transmission electron microscopy images of Afp; which was heterologously expressed in *E. coli* and later purified [41]. The *S. entomophila* Afp gene cluster comprises 18 open reading frames, encoding the structural proteins and putative effector toxins that are responsible for anti-feeding activity in grass grub larvae [41,42]. Furthermore, the A-T content in afp17 and afp18 genes is higher than that in other Afp genes, indicating that these two Afp genes are acquired by horizontal gene transfer, which is believed to be responsible for the acquisition of the DNA encoding a cytotoxin from within a region of typical nucleotide content in the phage region of pathogenic bacteria [40]. Recently, Desfosses et al. reported the high-resolution structure of the entire Afp particle in the extended state [12]. The extended Afp is a 110 nm-long bullet-shaped particle built up by a helical trunk with a conical cap at the apical end and a flat base with a mobile fibre network at the proximal end [12]. The final atomic model of extended Afp contains 11 different proteins [12]. The tail fibres formed by Afp13 were not well resolved but the similarity of the amino acid sequence of Afp13 and the fibre proteins of adenoviruses suggested that the host specificity of Afp is evolutionarily related to viruses targeting eukaryotic cells [10].

*Photorhabdus* virulence cassettes (PVCs) produced by *Photorhabdus* species are another model of gram-negative bacterial eCISs having an insecticidal activity (Figure 2) [43]. Bacteria belonging to *Photorhabdus* exist in a symbiotic partnership with entomopathogenic nematodes of *Heterohabditis* sp. [44]. *Photorhabdus* bacteria are delivered into the haemocoel of the insect after regurgitation from the nematode and subsequently bioconvert the insect tissues into a dense soup of *Photorhabdus* bacteria, which acts as a food source to support nematode replication [45]. The *Photorhabdus* genome encodes a diverse repertoire of virulence genes encoding protein toxins, proteases and lipases for combating diverse hosts, which can be found in chromosomally encoded pathogenicity islands [46]. Several classes of *Photorhabdus* insecticidal toxins have been characterised, such as toxin complexes, PirAB toxins, Mcf toxins and PVCs [47]. PVC is a highly distinct toxin delivery system representing operons of 16 conserved structural and synthetic genes [8]. This operon has two putative effector genes, PAU_03337 and PAU_03332 (Pnf). Pnf is a homologue of the active site domain of the *Yersinia* CNF2 (Cyto Necrosis Factor 2) toxin, which has a small GTPase deamidase and transglutaminase activity [45]. Vlisidou et al. showed that the Pnf effector is physically associated with the PVC needle complex which perforates the target cell membrane and exerts CNF2-like transglutamination and deamidation effects on purified small GTPases [45]. In 2019, Jiang et al. reported the cryo-EM structure of an intact PVC from *P. asymbiotica* [8]. PVC shows a simplified T4 phage tail-like structure containing a hexagonal baseplate complex with six fibres and a capped 117 nm sheath-tube trunk [8].

Metamorphosis-associated contractile structure (MAC) from *Pseudoalteromonas luteoviolacea* represents another eCIS group with distinct structural properties and biological functions [33]. Bacteria from the genus *Pseudoalteromonas* are commonly isolated from marine water, sediment, biofilms, or marine eukaryotes [48]. *P. luteoviolacea* strain HI1, isolated from a marine biofilm, triggers metamorphosis of marine tubeworm *Hydroides elegans*, which is a model for the study of bacteria-induced invertebrate metamorphosis [48]. In the vicinity of the *P. luteoviolacea* genes identified as essential for the induction of *H. elegans* metamorphosis, Shikuma et al. identified a cluster of open reading frames predicted to encode components of phage tail-like structures [33]. The genes were named MAC genes, and MAC particles were found to be responsible for metamorphosis (Figure 2). MACs are likely to be released through cell lysis; extracted and exogenously added MACs mediate the metamorphosis of *H. elegans*. Surprisingly, MACs are self-assembled intracellularly and expand as an ordered array upon cell lysis, which is mediated by the endolysin/holin system encoded in the MAC gene cluster [33]. A typical MAC array contains approximately 100 individual phage-like tails. MACs are oriented with outward baseplates with filamentous structures, probably tail fibres, emanating from the baseplates and appearing to connect individual MACs to each other [33]. Two years later, MACs were also shown to induce metamorphosis in *H. elegans* through the following different ways: (i) inducing larval settlement, (ii) initiating cilia loss and activation of metamorphosis-associated transcription, and (iii) signalling through p38 and c-Jun *n*-terminal kinase MAPK pathways, leading to morphological changes [49]. Among the six genes identified to be essential for inducing metamorphosis, the deletion of JF50_12615 (Metamorphosis-inducing Factor 1, Mif1) completely abolished the inducing effect of MACs [9]. Notably, the Δ*mif1* mutant produced MACs with no discernible density inside the tube lumen of MAC particles, while the wild-type counterpart exhibited repeating packets of density inside the tube, suggesting the presence of a potential cargo in the wild-type MACs [9]. Together with the results of mass spectrometry, Mif1 was suggested to be filled in the inner tube lumen of wild-type MACs [9]. In addition, JF50_12605 was found to be important for localising Mif1 within the MAC complex and interacting with Mif1, while the interaction between Mif1 and the MAC tube was found to be weak or absent [9]. Mif1 induced metamorphosis when purified and internalised into *Hydroides* cells by electroporation; therefore, the Mif1 filled in the tube lumen of MACs is hypothesized to be injected into the target cell with a tube-spike complex and exert its metamorphosis-inducing activity [9]. The function of Mif1 is currently unknown because of the absence of known conserved protein domains in Mif1. In addition to Mif1, the MAC-associated toxic effector protein JF50_12610 (Pne1) with nuclease activity has also been identified [50]. Although Pne1 is not required by MAC to induce tubeworm metamorphosis, MAC-mediated translocation of the effector protein kills insect cells and murine macrophages (Figure 2).

Notably, in some cases, eCISs are retained in the cytoplasm and are associated with the cell membrane. In 2017, Böck et al. presented a set of cryo-EM images showing T6SS-like nanostructures localised at the cell membrane of *Amoebophilus asiaticus*, an obligate intracellular bacterial symbiont of amoebae [34]. The *Amoebophilus* developmental and infection cycle in *Acanthamoeba* comprises the following stages: uptake, phagosome residence, phagosome escape, differentiation into rod-shaped cells and replication, re-differentiation into coccoid cells, and exit [51]. Notably, host infection rates and red blood cell haemolysis were positively correlated with the expression of the nanostructure [34]. As T6SS-mediated haemolytic activity has also been reported in other bacteria [24], this finding would suggest the functional similarity between typical T6SSs and T6SS-like nanostructures of *A. asiaticus* [34]. The authors also found that any contact site between the phagosome membrane and the outer membrane of the bacterium showed the presence of T6SS-like nanostructures [34]. These results suggest that the nanostructures mediate interactions with host membranes and may participate in phagosome escape. Surprisingly, despite their distinct subcellular localisation, the structural proteins of these nanostructures are more closely related to eCISs than T6SS [6,34]. Although they were described as a subtype of T6SS (T6SS*^iv^*) in the literature [34], the study strongly suggests that eCISs have evolved at least two ancestor groups with extracellular or intracellular localisation (Figure 2). Moreover, intracellular localisation of eCIS-related nanostructures may have some implications for rhapidosomes, which are tubular nanostructures that have been found in the cytoplasm of a variety of bacteria and algae [6,7,52,53,54]. Although the action mechanism of rhapidosomes is unclear, some of these nano-sized tubules show toxicity to mice, suggesting that they may play a role in animal–bacteria interactions [53]. As currently known, eCIS-related nanomachines can mediate the animal–bacteria interactions by various mechanisms (Figure 2), suggesting the potential functional and/or evolutionary relevance of rhapidosomes and eCISs [7].

#### 4.2.2. Gram-Positive Bacterial eCISs

Despite the recent progress in understanding the biological functions of eCISs in gram-negative bacteria, little is known about gram-positive bacterial eCISs. Comprehensive bioinformatics analyses of eCIS genes conserved among diverse microorganisms have revealed a broad existence of these genes in gram-positive as well as gram-negative bacteria [10]. Actinobacteria is a class of gram-positive bacteria in which eCIS genes are highly conserved [6,8,10]. Notably, eCIS gene clusters are conserved in 94 out of 116 complete genomes of *Streptomyces* species [10], suggesting the biological importance of eCIS genes in filamentous bacteria. *Streptomyces* species are known for their unique life cycle involving morphological differentiation associated with their ability to produce therapeutic antibiotics [55]. Their life cycle starts with the germination of a spore and the growth of the germ tube by apical tip extension [55]. Aerial mycelia extend into the air from these tubes and ultimately form spores [55]. In *S. coelicolor*, an extensively studied model of *Streptomyces*, eCIS-related genes are regulated by *bldA* that encodes UUA-tRNA^Leu^ translating the TTA codon. As this codon is very rare in the *Streptomyces* genome, which has a high G + C content, *bldA* can regulate the translation of genes containing the TTA codon [56,57]. Notably, many genes essential for such morphological differentiation are regulated by *bldA* [56,57]. Therefore, deletion of *bldA* leads to a phenotype that lacks aerial mycelia and spores [56]. In addition, *bldA* also acts as a trigger for secondary metabolite production, as many gene clusters responsible for secondary metabolite production contain TTA codons [56,57,58]. These observations collectively suggest pleiotropic and essential roles of *bldA* in *Streptomyces* life cycle.

It has been shown that the expression of the eCIS-related gene cluster in *S. coelicolor* is regulated by *SCO4263* encoding a cluster-specific LuxR-type transcriptional regulator [59,60]. As *SCO4263* contains the TTA codon, its expression is completely dependent on bldA; therefore, the eCIS-related gene cluster is regulated by *bldA* in *S. coelicolor* [59,60]. Although this previous observation suggests the biological importance of eCIS-related genes in the *Streptomyces* life cycle, the deletion mutants for the eCIS-related genes are phenotypically normal in terms of morphological differentiation and antibiotic production [59,60]. Due to the lack of apparent phenotypic changes in the deletion mutants, the biological significance of the highly conserved eCIS-related genes in *Streptomyces* has been unclear for over 15 years [59,60].

**Figure 3:**
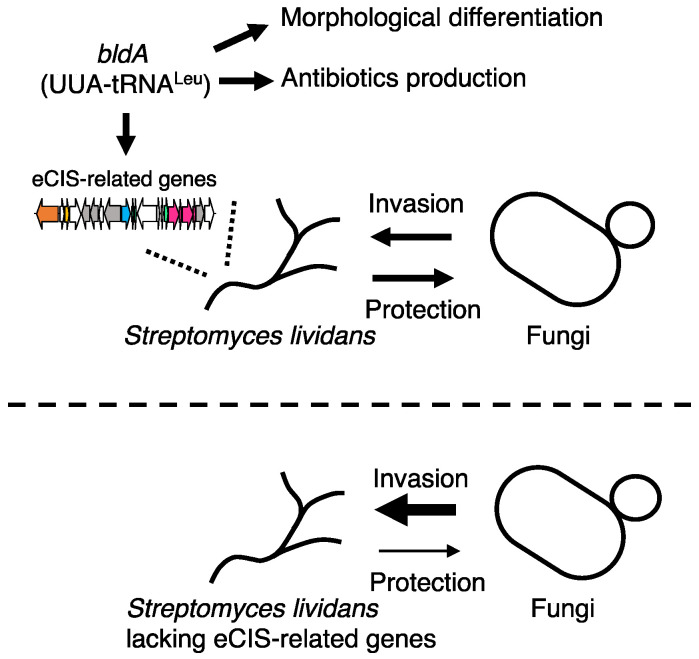
eCIS-related genes affect microbial interactions between *Streptomyces* and fungi.

Recently, it has been shown that *bldA*-dependent eCIS-related genes affect microbial interactions between fungi and *Streptomyces lividans*, a close relative of *S. coelicolor* [61] (Figure 3).

Schematic models indicating the competition between *Streptomyces lividans* and fungi mediated by eCIS-related genes. In *S. lividans*, eCIS-related genes are regulated by *bldA*, which also regulates morphological differentiation and antibiotic production in the bacterium. The arrows denoted as “Invasion” and “Protection” indicate the invasion of fungal cells into *S. lividans* colony and the resistance of *S. lividans* colony to the fungal invasion, respectively. The arrow with the thin line in the bottom illustration indicates that *S. lividans* mutant colony is less resistant to fungal invasion than the parental strain, and the arrow with the thick line indicates that fungal cells can severely invade the *S. lividans* colony.

The colonies of the deletion mutants of *S. lividans* for the eCIS-related genes are more severely invaded by fungi, such as *Saccharomyces cerevisiae* and *Schizosaccharomyces pombe*, than the parental strain [61] (Figure 3). The eCIS-related gene cluster is almost completely conserved among *S. lividans* and *S. coelicolor*, and gene deletion experiments confirmed that the *bldA*-dependent regulation of eCIS-related genes is a common mechanism among these species [61]. In addition, transmission electron microscopy images show that the products of these genes are phage tail-like nanostructures, which are similar to known gram-negative bacterial eCISs [45,61]. Based on these observations, the phage tail-like nanostructures found in *S. lividans* and *S. coelicolor* have been named *Streptomyces* phage tail-like particles (SLPs) [61] (Figure 3). Microscopic and transcriptional analyses demonstrated that SLP expression in *S. lividans* was upregulated upon co-culture with fungal competitors, further suggesting that SLP genes contribute to microbial competition between *Streptomyces* and fungi [61]. Although the roles of these eCIS-related genes in microbial interactions remain to be elucidated, further investigation will clarify how SLP confers selective advantages to *Streptomyces* species, which are ubiquitously found in densely populated polymicrobial communities, such as soil [62].

## 5. Discussion

Phage tail-like nanomachines have the potential to be applied as delivery tools for therapeutic purposes. Historically, phage particles have been recognised as excellent candidates for drug or gene delivery. Phage particles can be genetically engineered and tailored using phage libraries, as has been widely reviewed [63,64,65]. The applications of modified phage particles range from drug delivery nanomachines targeting pathogenic bacteria and cancer cells to collagen fibre-mimicking nanomaterials mediating the formation of artificial bones [66]. eCISs are functionally distinct from the phages used for medical applications because all known gram-negative bacterial eCISs are specified for protein translocation. The mechanism by which cargo proteins are selected and interact with eCIS particles is largely unclear [8,9,12] and uncovering these mechanisms may facilitate the development of modified eCISs with diverse functionalities. In addition, comprehensive genomic and functional analyses of potential eCIS effector proteins conserved among diverse classes of bacteria have recently been conducted [67]. Although the functional diversity and target specificity of the proposed eCIS effectors remain to be elucidated, collecting eCISs from various environments will facilitate the application of eCISs as promising nanoparticles targeting various types of cells, both eukaryotic and prokaryotic. As previous studies have suggested that proteinaceous effectors may be located inside the tube lumen of eCISs and ejected from the tube complex, possibly in a surface-charge-dependent manner [8,9,12], understanding the biochemical features of the effectors is likely to be key to altering eCIS functions [9]. The target specificities of currently known eCISs to eukaryotic cells can also be attributed to the tail fibres, which may have originated from adenovirus [8,10], and, therefore, target specificities may also be altered through exchange and/or mutation of the target recognition domains of the tail fibre. As mentioned in the previous reports [8,9,50], successfully engineered eCISs can potentially be used in biotechnological applications as a system to inject effector proteins with various biological functions, such as antibacterial and immunomodulatory activities, to the targeted cells. Moreover, I here suggest that they are safer and may be used for delivery purposes in clinical settings, because eCISs resemble phage tails but are not infectious and replicative, because of the absence of the head component [63].

Furthermore, understanding the ways in which phage tail-like nanomachines confer benefits to the producer cells would also be important to improve their applicability as versatile nano-sized delivery systems. The functions of eCISs are likely to be closely associated with the biological interactions of their hosts in their natural settings, as shown in the cases of Afp, PVC and MAC. However, the biological functions of eCISs in the vast majority of bacterial species that have eCIS-related genes in their genomes are largely unknown and must be explored. As these bacteria are widely distributed in natural environments, such as soil and sea, uncovering the mechanisms by which eCIS-related genes contribute to the fitness of the host bacterium experiencing various kinds of biological interactions and stresses would lead to the further discovery of the diverse functions of eCIS and offer insights into their use in clinical practice. In addition to the modification of eCIS functions, these ecological approaches will be necessary to expand the repertoire of the promising eCIS functions and overcome the current limitation of using eCISs as biological tools.

Finally, I would like to discuss the relation of eCIS with membrane vesicles (MVs), a major class of nanoparticles ubiquitously produced by bacteria. MVs are nano-sized spherical particles typically comprised of lipids, proteins, nucleic acids and other biomolecules derived from cells. For decades, various biological functions of bacterial MVs have been revealed. Bacterial MVs are involved in nutrient acquisition, horizontal gene transfer, antibiotic resistance and cell-cell communications [1,68,69]. MV functions depend on their biochemical composition, which can be determined by the mechanism of MV formation, and is a widely accepted fact [1,2,68,69]. Compositions of bacterial MVs are closely associated with those of cell membranes. In gram-negative bacteria in which the cell envelope is composed of two cellular membranes, outer MVs derived from the outer membrane are mainly released from the cells under normal culture conditions [1,68,69]. Additionally, Toyofuku and colleagues have proposed the prophage-mediated mechanism of MV formation, called explosive cell lysis, in gram-negative bacteria [70]. In this mechanism, DNA-damaging stress triggers the expression of lytic genes encoded in the prophage region of the *Pseudomonas aeruginosa* genome, resulting in cell lysis, with shattered cellular membrane fragments subsequently rounding up and forming MVs [70]. Moreover, the phage-mediated mechanism of MV biogenesis has also been found in gram-positive bacteria and mycolic acid-containing bacteria [71,72]. In this case, upon the onset of lytic processes, the cytoplasmic membrane of cells protrudes through the holes in the peptidoglycan layer that are formed by endolysin, which eventually leads to MV formation [71,72]. Considering that these viral lytic mechanisms are also involved in the release of eCISs and tailocins from cells [27,33], the production of these phage tail-like nanomachines could be an alternative route of cell lysis-mediated MV formation. Interestingly, such MVs produced by cell lysis-mediated mechanisms contain abundant cytosolic components, such as plasmids, genomic DNA and viral DNA [1], suggesting possible physiological roles of DNA-containing MVs in the transfer of phage-related genetic materials between bacterial cells [73,74,75]. Given the abundance of MVs in natural environments [76] and the accumulating evidence indicating the generality of phage-mediated MV production across bacterial species [70,71,72], bacterial MVs generated by cell lysis may have, in part, contributed to the spread and the diversification of phages and phage-related nanostructures, and ultimately the rise of eCISs.

Also, one should note that MVs and other nanoparticles derived from bacterial cells may cause problems in eCIS function analysis. As these nanoparticles are of similar sizes and may be co-precipitated by ultracentrifugation, which is a standard method to isolate phage tail-like nanostructures and MVs [73], the contamination of eCIS solutions with MVs and other nanoparticles, such as bacteriocins and phage particles during purification procedures [33], may interfere with the functional/structural analyses of eCISs and vice versa. The development of efficient methods for gene deletion/downregulation in the bacteria of interest or highly selective methods to separate these nanoparticles are required to circumvent this problem and facilitate further clarification of functions and ecological significance of these phage-related bacterial nanoparticles, especially in non-model species.

## 6. Conclusions and Future Directions

This review summarises the current knowledge on bacterial phage tail-like nanostructures with interesting functions, such as mediating various interactions between the producer bacteria and other organisms. These nanostructures variously benefit the producer bacteria and, in some cases, their host eukaryotes. These observations suggest that the phage-related genes have co-evolved with the producer microorganisms and have been maintained in their genomes by conferring them ecological fitness. It is often assumed that bacterial phage tail-like nanostructures have originated from prophages that lost their capsid genes and the ability to replicate. Most phages possess tails that provide them with a tremendous evolutionary advantage [77]; therefore, bacteria may have taken advantage of this system by incorporating the genes encoding phage tail into their genome to form phage tail-like nanomachines, including tailocins, T6SS and eCISs. The structural comparison of the contractile phage tails, tailocins, T6SSs and eCISs, also suggests that these nanomachines have evolved from a common cellular ancestor [34,78]. Therefore, the evolutionary history of these nanomachines remains an open question. In addition, eCISs are widely distributed among prokaryotes, but their biological functions are largely unknown. Discovering new eCISs and elucidating the functional and structural diversity of eCISs will lead to further understanding of the biological interactions involving microorganisms and provide insights into using eCISs to develop novel biological tools.
